# The Interaction Between Corn Starch and Xanthan Gum in Formulating Heat-Induced Emulsion Gels for Animal Solid Fat Mimetics

**DOI:** 10.3390/foods15091568

**Published:** 2026-05-02

**Authors:** Yuanqi Lv, Xiying He, Tingting Tang, Han Cui, Tingwei Zhu, Yujie Su, Guanhao Bu, Lilan Xu

**Affiliations:** 1 College of Food Science and Technology, Henan University of Technology, Zhengzhou 450001, China; 2 Chongqing Key Laboratory of Economic Plant Biotechnology, College of Landscape Architecture and Life Sciences, Chongqing University of Arts and Sciences, Chongqing 402160, China; 3 School of Food Science and Technology, Jiangnan University, Wuxi 214122, China; 4 Jiangxi Key Laboratory of Natural Products and Functional Food, Jiangxi Agricultural University, Nanchang 330045, China

**Keywords:** emulsion gel, xanthan gum, starch, solid fat replacer, gel properties

## Abstract

To mitigate health risks associated with animal solid fats, this study developed a heat-induced emulsion gel using corn starch and xanthan gum (XG) as the matrix. The effects of the oil-to-water ratio (20–40%) and XG content (0.1–0.5%) on gel properties were systematically investigated. Results suggested a significant two-way interaction (*p* < 0.05) between the oil–water ratio and XG content, which jointly optimized the three-dimensional network structure. The resulting gel (O40-XG0.5) exhibited rheological and textural properties—including an increased storage modulus (G′), hardness of 2420.74 g, and springiness of 0.97, which closely matched those of pork backfat. Microstructural and low-field NMR analyses suggested that XG may stabilize the oil–water interface via its amphiphilic nature and may form hydrogen bonds with starch, which could enhance the water/oil holding capacity and cooking stability (i.e., reduced oil exudation). This research offers a potential theoretical basis and technical pathway for developing plant-based solid fat replacers.

## 1. Introduction

Animal solid fats play important roles in foods, such as forming stable crystal network structures to improve product stability, and providing lubrication to enhance food texture [1]. However, due to their stable nature, slow metabolism in the body, and tendency to accumulate, they increase the risk of diseases. For instance, high levels of saturated and trans fatty acids can lead to dyslipidemia and cardiovascular abnormalities [2]. Therefore, developing solid fat replacers is crucial for optimizing industrial production and improving the sustainability of the modern food supply as well as consumer health.

Current methods for preparing solid fat replacers mainly include emulsion gels, oleogels, and bigels. Emulsion gels are defined as emulsions possessing gel-like network structures and solid-like textural properties [3]. The preparation of barley milk gels from barley flour containing cereal β-glucan blended with crude oil and water provides a method for developing oil-in-water emulsion gels as potential solid fat replacers, confirming the feasibility of emulsion gels to mimic solid fats [4]. Olive oil-in-water emulsion gels fortified with pea protein and guar gum as synergistic formulations for solid fat replacers also exhibited excellent rheological and gelling properties and a strong anti-phase separation ability, and effectively simulated natural solid fats [5]. Oleogels are defined as semi-solid fat-like systems formed by the self-assembly of gelators into a network structure that entraps liquid vegetable oils [6]. In frankfurters, partial replacement of animal fat with oleogels structured with monoglycerides and phytosterols resulted in products with a stronger oleogel network, higher hardness, and greater gel strength [7]. Bigels are defined as biphasic systems made by shearing and mixing oleogels and hydrogels at a certain temperature, possessing good stability and encapsulation properties [8]. Bigel systems constructed with xanthan gum and gelatin to simulate solid fat showed good thermal stability, resistance to deformation, and elastic recovery ability [9]. However, the application of oleogels faces challenges such as incompatibility with the texture of certain foods, and bigel technology is relatively difficult to construct, which limits its industrial development to some extent [10]. Therefore, considering industrial production, consumer health, and sensory evaluation, this study selected the emulsion gel approach to prepare solid fat replacers.

Starch has good thermal gelling properties and can serve as a gelling agent in heat-induced emulsion gels. For example, heat-induced emulsion gels have been prepared using whey protein as an emulsifier and potato starch as a gelling agent [11]. Corn starch, composed of approximately 20% amylose and 80% amylopectin, possesses relatively excellent hot paste and cold paste stability, as well as strong gel strength and freeze-thaw stability. However, due to the relatively low amylose content in corn starch, polysaccharides are often added to improve its gelling properties. For instance, adding Mesona chinensis polysaccharide can increase the pasting viscosity of corn starch and promote the formation of a more ordered structure in the system, and the addition of pectin or sodium alginate can improve the rheological properties and gelling properties of corn starch [12]. Xanthan gum (XG) is a viscous extracellular polysaccharide synthesized by Xanthomonas campestris. Its molecular structure consists of linearly connected repeating units of D-glucose, D-mannose, D-glucuronic acid, acetate, and pyruvate. XG molecules have both hydrophilic and lipophilic groups, enabling them to reduce the interfacial tension at the oil–water interface and improve the stability of emulsion products. Although XG cannot form gels alone, it can act synergistically with other biopolymers to enhance gel strength [13]. Studies have shown that the addition of XG is very effective in reducing syneresis, results in the fastest lipid digestion rate, and also significantly increases the lipid digestibility and bioaccessibility in starch-based filled hydrogels, reducing cooking loss and decreasing hardness and cohesiveness [14].

The central hypothesis of this study is that xanthan gum, due to its amphiphilic nature and ability to form hydrogen bonds with starch, can synergistically interact with corn starch to stabilize oil-in-water emulsions, and that this synergistic effect is dependent on both the oil–water ratio and XG concentration. The specific novelty of this work lies in systematically mapping the interactive effects of these two variables on the gel properties, which has not been, to our knowledge, previously reported for corn starch-XG systems designed as animal fat mimetics.

## 2. Materials and Methods

### 2.1. Materials

Rapeseed oil was purchased from COFCO Fortune Food Co., Ltd. (Beijing, China). Soy lecithin (purity ≥ 95%) was purchased from Hebei Qiansheng Biotechnology Co., Ltd. (Shijiazhuang, China). Corn starch (amylose content 24.5% ± 1.2%, moisture content 12.5% ± 0.5%) was purchased from Jiangsu Guxiangyuan Food Co., Ltd. (Yancheng, China) Xanthan gum (XG, food grade, viscosity 1200–1600 cP for 1% solution at 25 °C) was purchased from Sinopharm Chemical Reagent Co., Ltd. (Shanghai, China). All chemical reagents were of analytical grade and purchased from Sinopharm Chemical Reagent Co., Ltd.

### 2.2. Emulsion Preparation

Soy lecithin was dissolved in a small amount of ethanol. After the ethanol evaporated, rapeseed oil was added, with a mass ratio of rapeseed oil to soy lecithin of 10:1. Complete removal of ethanol was verified by weighing the lecithin–oil mixture before and after evaporation at 60 °C under constant stirring until constant weight (weight change <0.5% over 10 min), ensuring no residual solvent remained. The mixture was stirred at room temperature for 1 h to completely disperse the soy lecithin in the oil phase. Then, distilled water was added, with mass ratios of oil phase to water phase of 20%, 30%, or 40% (*w*/*w*, water phase basis). The oil-to-water ratio is expressed as the mass ratio of rapeseed oil to distilled water (e.g., 20% means 20 g oil per 100 g water). The total mass of the emulsion was not kept constant; instead, the oil phase mass was varied while the water mass was fixed at 100 mL (equivalent to 100 g), as shown in Table 1. The mixture was stirred for another 1 h, followed by high-speed shearing (16,000 rpm, 10 min) to prepare the emulsion. Then, based on the water phase mass, corn starch (40%, *w*/*w*, water phase basis) was added and stirred for 1 h to allow complete starch swelling. Subsequently, XG (0.1%, 0.3%, or 0.5%, *w*/*w*, water phase basis) was added and stirring was continued for 3 h to ensure uniform dispersion. A total of 9 samples were prepared, with specific formulations as listed in Table 1.

### 2.3. Rheological Properties of Emulsions

The rheological properties of the emulsions were measured using a dynamic rheometer with a 40 mm aluminum parallel plate geometry, and the gap was set to 1 mm. Frequency sweep tests were performed on the samples at 0.5% strain (within the linear viscoelastic region) over a frequency range of 0.1–100 rad/s [15]. Temperature sweeps were conducted at 1 Hz and 0.5% strain from 25 °C to 95 °C, with a heating rate of 5 °C/min. Changes in the storage modulus (G′) were recorded.

### 2.4. Emulsion Gel Preparation

Five grams of emulsion were transferred to a 10 mL beaker and sealed with plastic wrap. The samples were then heated in a water bath (95 °C, 30 min) to obtain the emulsion gels [16]. After heating, the gels were cooled to room temperature (25 ± 1 °C) for 1 h and then stored at 4 °C for 24 h to allow complete gel stabilization before texture and NMR measurements. Three independent batches were prepared on different days to assess batch-to-batch reproducibility.

### 2.5. Textural Properties of Emulsion Gels

The emulsion gels were subjected to texture profile analysis. The test used a P/36R probe, a trigger force of 5 g, a deformation of 50%, and pre-test, test, and post-test speeds of 2 mm/s [17].

### 2.6. Oil and Water Distribution in Emulsion Gels

A pulsed NMR analyzer was used to test the oil and water distribution in the emulsion gels, and the CPMG pulse sequence was used to collect the signals. The main parameter settings were as follows: resonance frequency 21 MHz, echo time 0.5 ms, waiting time 2500 ms, 8000 echoes, and 8 scans [18]. The Multi-Exp Inv analysis software (Niumag Inc., Shanghai, China) was used to fit the CPMG decay curves to obtain the transverse relaxation time (T_2_) and the corresponding peak area proportion (P_2_).

### 2.7. Cooking Properties of Emulsion Gels

The emulsion gels were baked in an oven at 160 °C for 20 min, and their oil and water exudation were observed.

### 2.8. Microstructure of Emulsion Gels

The emulsion gels were cut into thin slices (5 mm × 5 mm × 1.5 mm), rapidly frozen using liquid nitrogen, and then dried in a freeze dryer. Finally, the microstructure of the emulsion gels was observed using a cold field emission scanning electron microscope [19].

### 2.9. Statistical Analysis

All measurements were conducted in triplicate, and the data were expressed as the mean ± SD. The effects of the oil-to-water ratio, xanthan gum content, and their interaction term on the gel properties were analyzed by two-way ANOVA using SPSS 20.0 (SPSS Inc., Chicago, IL, USA), and the differences among means (*p* < 0.05) were compared with Tukey’s multiple comparison.

## 3. Results and Discussion

The results of two-way ANOVA (Table 2) showed that the oil–water ratio had significant effects on all textural and water distribution properties (*p* < 0.05 for all parameters). The XG content also significantly affected most parameters, except for springiness (*p* = 0.792). In addition, the interaction of the oil–water ratio and XG content significantly affected hardness, cohesiveness, chewiness, resilience, and water-holding capacity (*p* < 0.05), indicating that XG content and oil–water ratio have a synergistic effect on the macroscopic properties of the emulsion gel.

### 3.1. Rheological Properties of Emulsions

#### 3.1.1. Frequency Sweep

**Figure 1:**
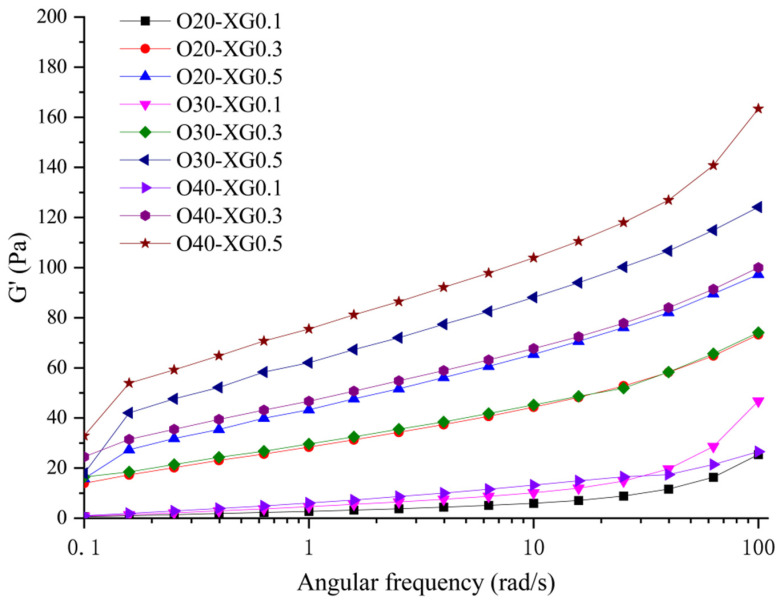
Evolution of G′ with oscillatory frequency in emulsions: Effect of XG content and oil–water ratio.

Figure 1 shows the variation of the storage modulus (G′) with angular frequency for different emulsions. As shown, the G′ values of all emulsions increased with increasing angular frequency, indicating an enhanced elastic response and good structural stability of the system under high-frequency conditions, exhibiting typical frequency-dependent rheological behavior. Further analysis showed a significant interaction between the XG content and oil/water phase ratio on G′. When the XG content was low (0.1%), changes in the oil phase ratio had no significant effect on G′. However, when the XG content increased to 0.3% or 0.5%, G′ increased significantly with an increasing oil phase ratio. On the other hand, under low oil–water ratio (20%) conditions, increasing the XG content had a limited effect on enhancing G′; whereas under high oil–water ratio (40%) conditions, G′ increased significantly with an increasing XG content. This indicates a synergistic effect between XG and oil phase ratio in regulating the elastic modulus of the emulsion [20]. A similar phenomenon has been observed in mayonnaise systems, where gel strength depends on both oil and XG concentration, with the elastic modulus and complex viscosity increasing with both [21].

From a mechanistic perspective, the enhancing effect of XG on G′ may be related to its molecular structure and interfacial behavior. The introduction of XG may increase the viscosity of the aqueous phase and competes with starch for water binding, which could lead to an increase in the effective starch concentration and enhanced intermolecular interactions, thereby potentially strengthening the elastic response of the system. It was hypothesized that XG, as an amphiphilic polysaccharide, may migrate to the oil–water interface and contribute to emulsion stabilization, possibly through droplet coating or the formation of flocculation bridges, as suggested by previous studies on similar systems [22]. However, direct visualization of these phenomena was not performed in this study. Furthermore, XG, as an amphiphilic polysaccharide, tends to migrate to the oil–water interface, stabilizing the emulsion droplets by coating them; it can also form flocculation bridges between droplets, further enhancing the viscoelasticity of the emulsion. Studies have shown that at high XG concentrations, its widespread distribution in the system not only increases the negative charge density on the droplet surface but also promotes the formation of a flocculated network [23]. Under high oil phase ratio conditions, the number of emulsion droplets and available binding sites for XG increase, raising the probability of bridging, which may contribute to the increase in the G′ value.

#### 3.1.2. Temperature Sweep

**Figure 2:**
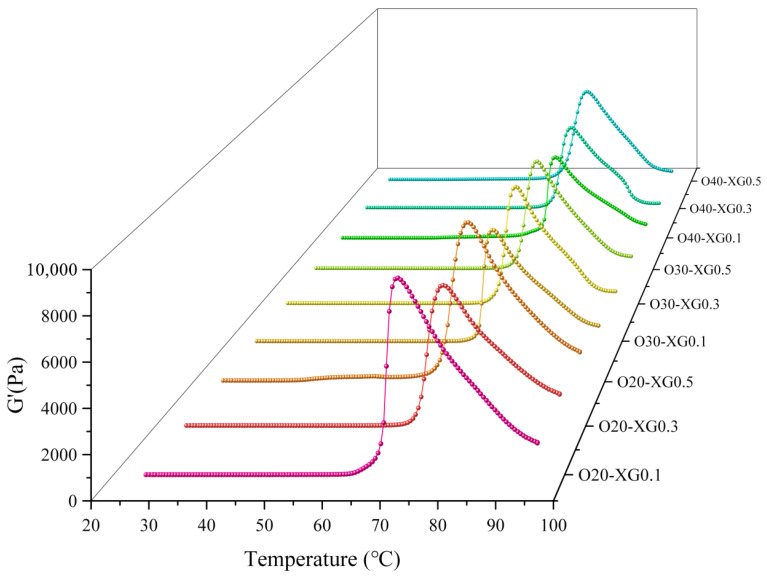
Evolution of G′ with temperature in emulsions: Effect of XG content and oil–water ratio.

To investigate the effect of temperature on the gelation behavior of the emulsions, temperature sweep analysis of their rheological properties was conducted in the range of 25 °C to 95 °C (Figure 2). The results showed that the storage modulus (G′) first increased and then decreased with increasing temperature, a change closely related to the starch gelatinization process. Specifically, G′ began to increase significantly at 65 °C, indicating irreversible water absorption and swelling of starch; in the range of 65–75 °C, G′ increased rapidly, marking the formation of the emulsion gel network; the subsequent decrease in G′ was attributed to the disruption of the gel cross-linked structure at high temperatures [24].

As the oil phase ratio increased, the peak G′ value of the system gradually decreased, mainly due to the inhibition of intermolecular cross-linking between starch molecules by the oil phase [23]. Further research found that the oil–water ratio and XG content had a synergistic regulatory effect on the peak G′. Specifically: at a 20% oil–water ratio, the order of peak G′ was O20-XG0.1 > O20-XG0.5 > O20-XG0.3; at a 30% oil–water ratio, it was O30-XG0.3 > O30-XG0.1 ≈ O30-XG0.5; and at a 40% oil–water ratio, it was O40-XG0.5 > O40-XG0.1 > O40-XG0.3. It is worth noting that the optimal conditions for peak G′ showed obvious ratio dependence, i.e., low XG content (0.1%) with low oil–water ratio (20%), medium XG content (0.3%) with medium oil–water ratio (30%), and high XG content (0.5%) with high oil–water ratio (40%) exhibited the best synergistic effects, respectively. 

Based on the above results, this study suggests a possible dual-stage mechanism by which XG enhances emulsion gel performance at a fixed oil–water ratio: at low concentrations, XG preferentially adsorbs at the oil–water interface, stabilizing the emulsion droplets and providing uniform support for the starch gel network. At high concentrations, however, excess XG disperses in the aqueous phase, increasing the effective starch concentration by water absorption and thickening, and promoting amylopectin rearrangement via hydrogen bond cross-linking, thereby strengthening the gel network. Especially in systems with an oil–water ratio not less than 30%, the increased interfacial adsorption capacity and the synergistic effect of particle coating significantly enhanced the elastic modulus of the system [22,25]. This finding offers a potential new theoretical perspective for explaining the role of polysaccharides in emulsion gels.

### 3.2. Gel Properties of Emulsions

#### 3.2.1. Textural Properties of Emulsion Gels

The gelation process of starch originates from the absorption of water, swelling, and rupture of its granules during heating, followed by the leaching of internal amylose, which forms a three-dimensional network structure through intermolecular interactions and rearrangement [26]. Both the oil–water ratio and XG content significantly (*p* < 0.001, Table 2) affected the textural properties of the emulsion gels, with a significant interaction effect (*p* < 0.001). As the oil phase ratio increased, the hardness, cohesiveness, chewiness, and resilience of the emulsion gels significantly decreased (Table 3). This was likely because the oil phase hindered the effective interaction between starch molecules, weakening the structural integrity and complexity of the gel network. For example, in plant-based pork skin prepared using components such as soy protein isolate and sweet potato starch, the oil phase ratio was also negatively correlated with gel hardness [27]. When the oil phase ratio was too high, the oil filled the gel pores, prompting starch molecules to self-aggregate rather than participate in the overall network construction and further weakening their gelling ability [28]. However, the increase in oil phase ratio improved the springiness of the gels to some extent, due to the filling of network voids by the oil, which enhanced the structural continuity [29].

Two-way ANOVA (Table 2) revealed that both the oil–water ratio and XG content significantly affected hardness, cohesiveness, chewiness, and resilience (*p* < 0.001), with significant interaction effects (*p* < 0.001). Regarding springiness, the oil–water ratio and the interaction exerted a significant effect, while the XG content did not (*p* > 0.05). Under the same oil phase ratio, as the XG content increased, the hardness, cohesiveness, chewiness, and resilience of the emulsion gels were improved. XG may not only enhance the interactions between starch molecules but also increase the effective starch concentration in the system through its high water-holding capacity, potentially promoting local cross-linking of amylose and the formation of the gel network. Furthermore, it is possible that hydrogen bonding between XG and amylopectin also contributes to the rearrangement of starch molecules, leading to a more stable network structure. In the presence of amylopectin, the self-aggregation behavior of starch molecules is suppressed, and instead, they form ordered connections with amylopectin through their linear segments. Such composite structures are further integrated into the gel network, enhancing the stability of the overall cross-linked system [30]. Especially under high oil phase ratio (40%) conditions, the increase in XG content further enhanced the gel springiness, which could be attributed to the accumulation of XG at the oil–water interface increasing the interfacial layer thickness [31]. It is worth noting that the emulsion gel with the O40-XG0.5 formulation most closely matched the textural properties of pork backfat reported in the literature, with a hardness of 2420.74 g and springiness of 0.97, compared to the reported values of 2222.03 g and 0.93, respectively [32]. However, a direct comparison with animal fat should be conducted in future studies. 

#### 3.2.2. Oil and Water Distribution in Emulsion Gels

The low-field NMR (LF-NMR) relaxation data for water distribution in the emulsion gels are shown in Table 4. The relaxation time T_2b_ (3–6 ms) corresponds to water tightly bound to macromolecules such as starch and XG; T_21_ (60–90 ms) represents water with restricted mobility within the gel network; and T_22_ (500–700 ms) reflects free water in the system. P_2b_, P_21_, and P_22_ represent the signal proportions corresponding to these three states, respectively.

Two-way ANOVA (Table 2) revealed that both the oil–water ratio and XG content significantly influenced the relaxation times and their proportions (*p* < 0.05). As the oil phase ratio increased to 30% and 40%, the T_2b_ and T_21_ relaxation times significantly shortened (*p* < 0.05), suggesting that under high oil phase conditions, oil droplets filling the pores restricted water migration, and more water molecules became tightly bound to XG and starch. At this point, the T_22_ signal appeared, and P_2b_ decreased while P_22_ increased, reflecting partial oil–water phase separation, with only part of the water remaining in a tightly bound state. 

After increasing the XG content, P_22_ significantly decreased (*p* < 0.01) at 30% and 40% oil phase ratios, suggesting that XG reduced the free water content through its strong hydrophilicity and possibly forming hydrogen bond cross-links with starch, thereby reducing the network pore size. Meanwhile, T_2b_ further shortened, indicating that XG effectively enhanced the water and oil holding capacity of the system. This result is consistent with the literature: XG can improve the water-holding capacity and mechanical strength of whey protein gels and reduce the proportion of free water in chestnut starch gels [19]. The mechanism could be mainly due to XG competing with starch for water binding, reducing the relative content of free water [33]. In summary, when the oil phase ratio is not less than 30%, the interfacial effect of XG is most significant, and the water carrying capacity of the gel network reaches its maximum.

### 3.3. Macroscopic Morphology and Cooking Properties of Emulsion Gels

**Figure 3:**
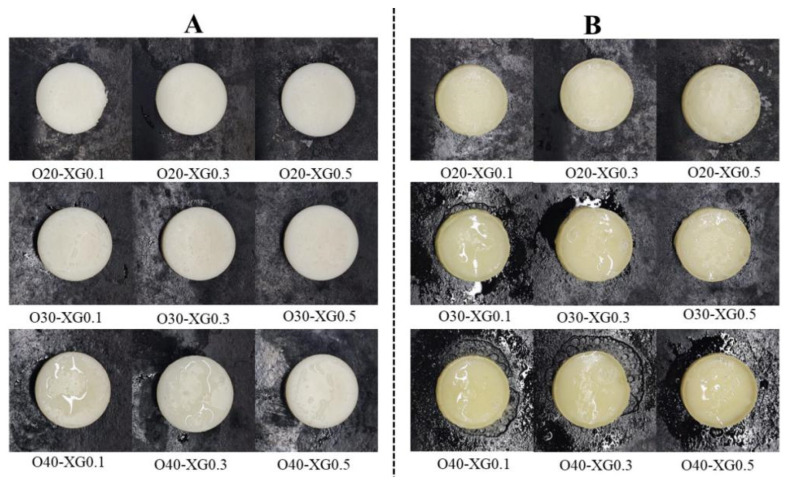
Morphological transition of emulsion gels induced by baking: untreated state (**A**) vs. post-baking state (**B**).

Figure 3A,B shows the macroscopic morphological changes of the emulsion gels before and after baking. As the oil phase ratio increased from 20% to 40%, the sample color gradually changed from milky white to light yellow, and surface oil exudation significantly increased (Figure 3A). This was likely due to the weakening of the gel network structure, leading to oil droplet aggregation and phase migration. After baking at 160 °C for 20 min, the samples with a 20% oil phase ratio still maintained a dry surface without obvious oil exudation. The samples with 30% and 40% oil phase ratios, on the other hand, showed significant oil separation, indicating that their thermal stability decreased with increasing oil phase.

At the same oil phase ratio, samples with a higher XG content exhibited lower oil exudation. XG may improve structural stability through a dual mechanism: on the one hand, it enhances the mechanical strength of the emulsion interfacial film, and on the other hand, it optimizes the starch gel network structure, which may help embed and immobilize the oil droplets [29,34]. It is worth noting that high XG content samples (such as O30-XG0.1, O30-XG0.3, and O40-XG0.5) showed controlled oil exudation after baking. This behavior is consistent with the typical thermal response characteristics of animal solid fats—no exudation before heating, but obvious oil separation after heating. Combined with the matching degree of textural properties, O40-XG0.5 was the closest to pork backfat in terms of key indicators such as hardness and springiness, suggesting its potential application as a solid fat replacer.

### 3.4. Microstructure of Emulsion Gels

**Figure 4:**
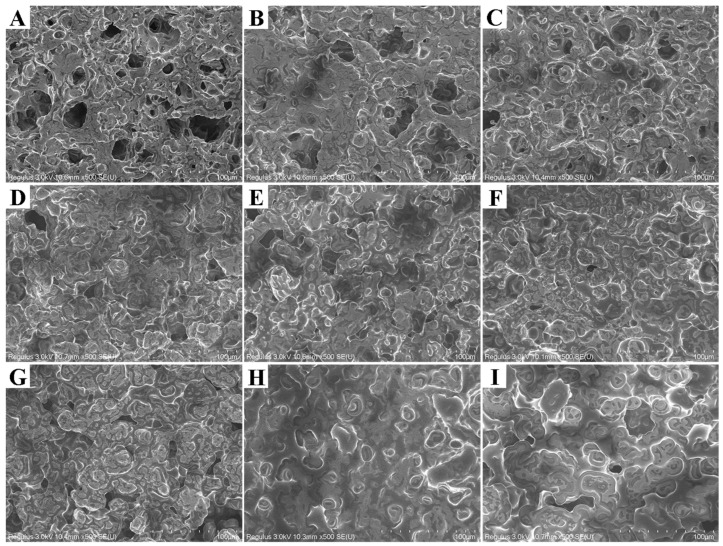
SEM micrograph of the emulsion gel at 500× magnification (O20-XG0.1, (**A**); O20-XG0.3, (**B)**; O20-XG0.5, (**C**); O30-XG0.1, (**D**); O30-XG0.3, (**E**); O30-XG0.5, (**F**); O40-XG0.1, (**G**); O40-XG0.3, (**H)**; O40-XG0.5, (**I**)).

Figure 4 shows the microstructure of emulsion gels with different formulations. When the oil phase ratio was 20%, the gels exhibited a typical porous network structure, presenting a honeycomb-like morphology with uneven pore size distribution, and the pores were interconnected to form a dense three-dimensional network (Figure 4A–C). This structure is consistent with findings in OSA starch-based emulsion gels and plant-based imitation pork skin products [27].

When the oil phase ratio increased to 40%, the gel matrix changed significantly with a marked reduction in pore number (Figure 4G–I), a structural transformation attributed to the pore-filling effect of oil droplets, consistent with findings in rice starch-pectin systems [29]. It is worth noting that the high oil phase ratio damaged the starch network, forming a large number of starch granule fragments (Figure 4G–I). One possible explanation is the hindered cross-linking of amylose; these fragments embedded in the gel will have an important impact on the textural properties [35]. As the oil phase ratio increased, the microstructure gradually changed from rough and uneven to uniform and smooth, indicating that the oil phase participated in building the network and produced a coating effect on the internal structural surface [24]. Among them, the samples with a 30% oil phase ratio presented the most uniform and fine matrix structure (Figure 4D–F), while the 40% samples showed more starch granules/fragments and obvious oil phase.

At a fixed oil phase ratio, the effect of increasing the XG content on the gel matrix showed concentration dependence. At 20% and 30% oil phase ratios, the increase in XG led to a reduction in pores (compare Figure 4D–F), which stemmed from the hydrophilic nature of XG causing water competition, reducing the free water content in the system, and the syneresis effect intensified with increasing colloid concentration. In the 40% oil phase samples, the protective effect of XG on starch granules was particularly significant: compared with the accumulation of fragments in O40-XG0.1 (Figure 4G), the starch granule morphology in O40-XG0.3 and O40-XG0.5 was more complete (Figure 4H,I), and the formation of a smooth oil film was observed in O40-XG0.5 (Figure 4I) [33]. This phenomenon could be attributed to the coating effect of XG on the starch surface, consistent with studies on the coating effect of xanthan gum on cassava starch [24]. XG may not only protect the integrity of starch granules but also enhance the structural stability of the system by strengthening the intermolecular interactions between starch molecules.

### 3.5. Correlation Analysis Among Rheological, Textural and Water Distribution Properties

To quantitatively evaluate the relationships between gel elasticity, texture, and water mobility, Pearson correlation analysis was performed. The storage modulus at the highest tested angular frequency (G′ at 100 rad/s) was used as the rheological indicator, together with texture profile analysis parameters and LF-NMR water distribution data (P_21_ and P_22_). Only samples with detectable P_22_ were included in the correlation analysis involving free water (n = 6).

**Figure 5:**
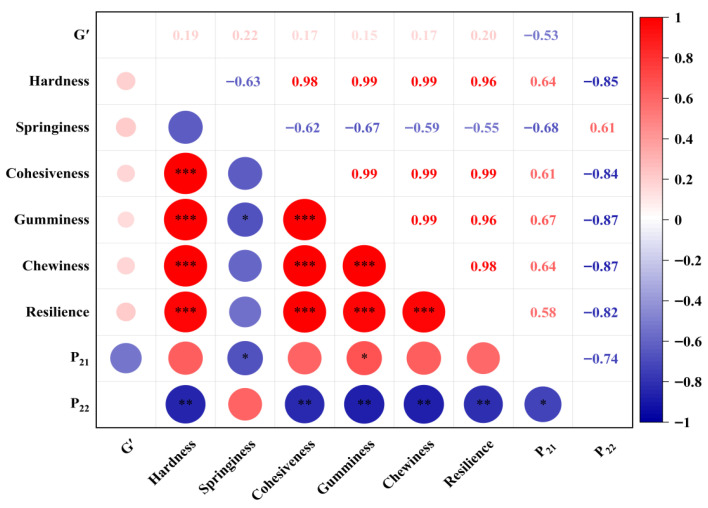
Pearson correlation heatmap of rheological, textural, and water distribution parameters.(* *p* < 0.05, ** *p* < 0.01, *** *p* < 0.001).

As shown in Figure 5, G′ did not exhibit statistically significant linear correlations with any of the textural parameters or water distribution indices (*p* > 0.05 for all). For instance, the correlation coefficients between G′ and hardness, chewiness, P_21_, and P_22_ were 0.19 (*p* = 0.62), 0.17 (*p* = 0.66), −0.53 (*p* = 0.15), and 0.02 (*p* = 0.96), respectively. This lack of significant linear correlation is not unexpected, because G′ was measured on the emulsions before gelation, whereas texture and NMR were determined on the heat-induced gels after starch gelatinization and network formation. The gelation process (heating at 95 °C for 30 min) fundamentally alters the system’s mechanical and water-binding properties, which may decouple the pre-gel elastic modulus from the final gel characteristics.

Nevertheless, when the data are examined in a formulation-dependent manner (as discussed in Section 3.2.1 and Section 3.4), certain trends become evident. For example, under a fixed oil–water ratio, increasing the XG content consistently improved G′ (Figure 1), hardness, and chewiness (Table 3), while reducing free water (P_22_, Table 4) and leading to a more compact microstructure (Figure 4). These formulation-wise parallel trends suggest that G′ serves as a useful indicator of the emulsion’s structural strength before gelation, which indirectly influences the final gel properties through the same formulation parameters (oil–water ratio and XG content). In other words, the correlation is not linear across all samples but is captured by the synergistic effect of the two formulation variables, as confirmed by the two-way ANOVA interaction terms (Table 2). 

In contrast, a significant negative correlation was observed between hardness and P_22_ (r = −0.85, *p* = 0.0034), confirming that firmer gels contain less free water—a finding that aligns well with the microstructural observation that a denser network (e.g., O40-XG0.5) restricts water mobility. Additionally, hardness was strongly positively correlated with cohesiveness (r = 0.98, *p* < 0.0001) and chewiness (r = 0.99, *p* < 0.0001), which is expected as these parameters are derived from the same texture profile analysis.

Overall, while a direct linear correlation between the pre-gel G′ and final gel properties is not statistically significant in this dataset, the formulation-dependent trends and the significant interaction effects (Table 2) provide strong evidence that G′ and the final gel texture/water distribution are co-regulated by the oil–water ratio and XG content.

### 3.6. Discussion

**Figure 6:**
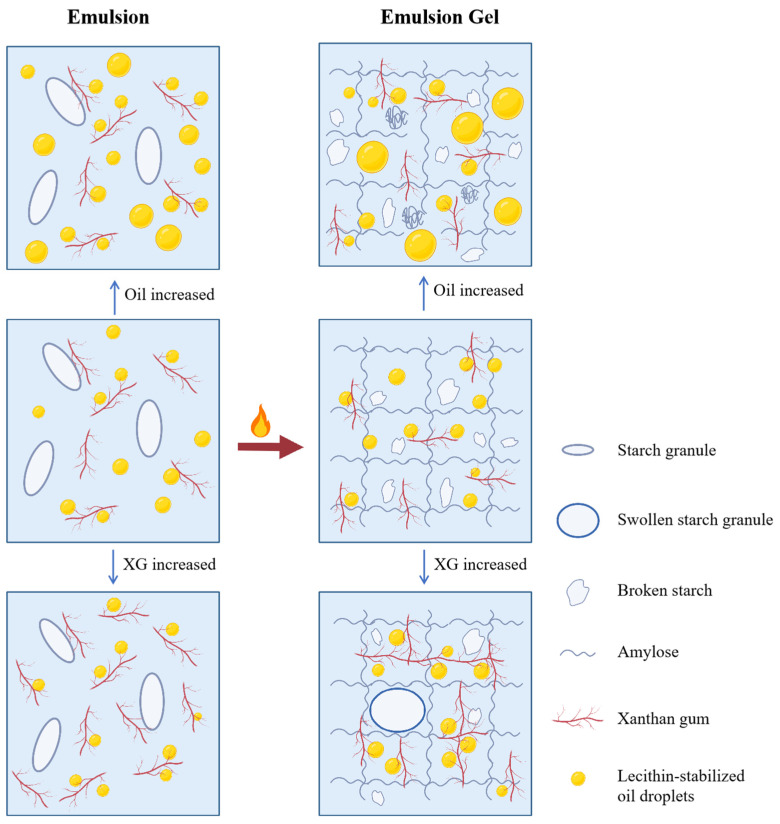
Mechanism diagram.

The experimental results suggest that the oil–water ratio and XG content significantly affect the properties of the corn starch-XG emulsion gels, with a significant two-way interaction between these factors. Figure 6 summarizes the possible relationships among formulation, structure, and properties observed in this study. Based on the data from rheology, texture, LF-NMR, and SEM, the following discussion offers a possible interpretation of the potential underlying links.

The oil–water ratio serves as a primary determinant of gel structure. A low ratio (20%) favors the formation of a starch-dominated porous network characterized by high hardness. As the ratio increases to 40%, the expanded oil–water interface demands greater stabilizer capacity, which in turn leads to partial phase separation, as evidenced by increased oil exudation and a higher proportion of free water [36]. Within this context, XG, an amphiphilic polysaccharide, is believed to adsorb at the oil–water interface and form a viscoelastic film [37]. At a low XG content (0.1%), emulsion stability remains limited. In contrast, when the XG content reaches 0.3–0.5%, it enhances aqueous phase viscosity, competes with starch for water, reduces interfacial tension, and provides steric hindrance [38]. Collectively, these effects promote a more uniform oil droplet distribution, a more robust gel network, and improved emulsion stability [39].

Temperature sweep analysis further reveals a three-stage gelation process: a slow modulus change between 25 and 65 °C, a sharp increase between 65 and 75 °C due to starch gelatinization, and a subsequent decline at higher temperatures indicating partial network disruption. The oil–water ratio also modulates the final texture: the 20% ratio yields a loose yet hard network, whereas the 40% ratio combined with 0.5% XG produces a denser network with lower hardness but higher springiness. Under sufficient XG conditions, oil droplets appear to act as active fillers that enhance network density [40]. Notably, the optimal formulation (O40-XG0.5) not only achieves textural properties comparable to pork backfat but also exhibits controlled oil exudation upon baking—a thermal response characteristic of animal solid fats. Together, these mutually reinforcing rheological, textural, and microstructural observations support a dual mechanism for XG: enhancing interfacial film strength while optimizing the starch gel network to embed and immobilize oil droplets. In summary, XG and starch synergistically modulate gel properties in an oil–water ratio-dependent manner. The optimal formulation (O40-XG0.5) achieves a favorable balance of rheological and textural properties alongside reduced oil exudation upon cooking.

## 4. Conclusions

This study suggests that heat-induced emulsion gels based on corn starch and xanthan gum (XG) can be formulated to simulate animal solid fat. The oil–water ratio (20–40%) and XG content (0.1–0.5%) exhibited a significant two-way interaction on gel properties. Specifically, combining a high oil–water ratio (40%) with a high XG content (0.5%) resulted in a dense. network structure. This O40-XG0.5 gel possessed textural properties (hardness 2420.74 g, springiness 0.97) that were comparable to literature-reported values for pork backfat, and showed reduced oil exudation after cooking. Rheological measurements suggested that XG was associated with increased storage modulus, and microstructural analysis revealed that XG contributed to a more compact network with fewer pores. It should be noted that this study did not provide direct molecular-level evidence for the proposed interaction mechanisms. Additionally, only one type of oil and starch source was evaluated. Future work should validate the optimized emulsion gel in real food systems and confirm the proposed interaction mechanisms using direct structural and interfacial analyses.

## Figures and Tables

**Table 1:** The formulation of the samples.

Sample	Soy Lecithin (g)	Rapeseed Oil (g)	Distilled Water (mL)	Corn Starch (g)	XG (g)
O20-XG0.1	2	20	100	40	0.1
O20-XG0.3	2	20	100	40	0.3
O20-XG0.5	2	20	100	40	0.5
O30-XG0.1	3	30	100	40	0.1
O30-XG0.3	3	30	100	40	0.3
O30-XG0.5	3	30	100	40	0.5
O40-XG0.1	4	40	100	40	0.1
O40-XG0.3	4	40	100	40	0.3
O40-XG0.5	4	40	100	40	0.5

**Table 2:** Significance values for the main effects of oil–water ratio (O), xanthan gum content (XG), and their interaction (O × XG) on gel properties of emulsion gels.

	Oil–Water Ratio (O)	XG Content (XG)	O × XG
Hardness	<0.001	<0.001	<0.001
Springiness	0.018	0.792	0.027
Cohesiveness	<0.001	<0.001	<0.001
Chewiness	<0.001	<0.001	<0.001
Resilience	<0.001	<0.001	<0.001
P_21_	<0.001	0.014	0.008
P_22_	<0.001	0.021	0.011

**Table 3:** Texture profile analysis of emulsion gels at different XG content and oil–water ratio.

Sample	Hardness (g)	Springiness	Cohesiveness	Chewiness (g)	Resilience
O20-XG0.1	3437.38 ± 154.97 ^c^	0.86 ± 0.08 ^bcd^	0.55 ± 0.01 ^b^	1627.64 ± 196.20 ^b^	0.27 ± 0.01 ^bc^
O20-XG0.3	4195.17 ± 39.28 ^b^	0.83 ± 0.08 ^bcd^	0.64 ± 0.06 ^a^	2209.92 ± 59.07 ^a^	0.35 ± 0.07 ^a^
O20-XG0.5	4579.52 ± 165.88 ^a^	0.77 ± 0.03 ^d^	0.65 ± 0.02 ^a^	2282.56 ± 42.41 ^a^	0.33 ± 0.01 ^a^
O30-XG0.1	1939.23 ± 94.65 ^f^	0.89 ± 0.02 ^abc^	0.43 ± 0.04 ^cd^	738.25 ± 70.10 ^d^	0.20 ± 0.02 ^de^
O30-XG0.3	3047.48 ± 166.40 ^d^	0.88 ± 0.06 ^abc^	0.56 ± 0.03 ^b^	1514.03 ± 80.74 ^b^	0.29 ± 0.04 ^ab^
O30-XG0.5	3424.58 ± 166.44 ^c^	0.80 ± 0.07 ^cd^	0.56 ± 0.03 ^b^	1532.21 ± 284.91 ^b^	0.28 ± 0.02 ^bc^
O40-XG0.1	1233.28 ± 59.11 ^g^	0.85 ± 0.02 ^bcd^	0.39 ± 0.01 ^d^	409.07 ± 21.25 ^e^	0.17 ± 0.01 ^e^
O40-XG0.3	2040.21 ± 153.61 ^f^	0.90 ± 0.02 ^ab^	0.46 ± 0.01 ^c^	856.42 ± 77.86 ^d^	0.23 ± 0.01 ^cd^
O40-XG0.5	2420.74 ± 198.65 ^e^	0.97 ± 0.02 ^a^	0.47 ± 0.05 ^c^	1083.03 ± 39.48 ^c^	0.23 ± 0.04 ^cd^

Note: Values are means ± SD (*n* = 3). Different superscript letters within the same column indicate significant differences (*p* < 0.05).

**Table 4:** Relaxation times and water proportions of emulsion gels.

Sample	T_2b_ (ms)	T_21_ (ms)	T_22_ (ms)	P_2b_ (%)	P_21_ (%)	P_22_ (%)
O20-XG0.1	5.54 ± 0.40 ^a^	86.98 ± 0.00 ^a^	–	8.12 ± 0.37 ^a^	91.88 ± 0.37 ^ab^	–
O20-XG0.3	5.54 ± 0.40 ^a^	86.98 ± 0.00 ^a^	–	8.18 ± 0.62 ^a^	91.82 ± 0.62 ^b^	–
O20-XG0.5	4.38 ± 0.79 ^ab^	75.65 ± 0.00 ^b^	–	7.74 ± 0.38 ^ab^	92.26 ± 0.38 ^ab^	–
O30-XG0.1	3.79 ± 0.57 ^bc^	75.65 ± 0.00 ^b^	639.56 ± 82.82 ^a^	6.96 ± 0.25 ^cd^	92.35 ± 0.29 ^ab^	0.69 ± 0.11 ^c^
O30-XG0.3	3.97 ± 0.91 ^bc^	75.65 ± 0.00 ^b^	603.04 ± 138.73 ^ab^	7.38 ± 0.16 ^bc^	91.97 ± 0.26 ^ab^	0.65 ± 0.10 ^c^
O30-XG0.5	4.22 ± 1.48 ^bc^	75.65 ± 0.00 ^b^	501.18 ± 64.98 ^b^	7.07 ± 0.32 ^cd^	92.34 ± 0.35 ^ab^	0.59 ± 0.12 ^c^
O40-XG0.1	3.45 ± 0.79 ^bc^	75.65 ± 0.00 ^b^	593.61 ± 39.96 ^ab^	6.45 ± 0.19 ^e^	92.20 ± 0.09 ^ab^	1.35 ± 0.13 ^a^
O40-XG0.3	3.08 ± 0.49 ^c^	68.26 ± 4.93 ^c^	536.27 ± 61.08 ^ab^	6.35 ± 0.26 ^e^	92.44 ± 0.31 ^a^	1.21 ± 0.09 ^ab^
O40-XG0.5	3.93 ± 0.54 ^bc^	73.18 ± 4.93 ^b^	516.29 ± 34.76 ^ab^	6.73 ± 0.14 ^de^	92.18 ± 0.26 ^ab^	1.10 ± 0.21 ^b^

Note: Values are means ± SD (n = 3). Different superscript letters within the same column indicate significant differences (*p* < 0.05).

## Data Availability

The original contributions presented in this study are included in the article. Further inquiries can be directed to the corresponding authors.
